# Water and beverage consumption among adults in the United States: cross-sectional study using data from NHANES 2005–2010

**DOI:** 10.1186/1471-2458-13-1068

**Published:** 2013-11-12

**Authors:** Adam Drewnowski, Colin D Rehm, Florence Constant

**Affiliations:** 1 Université Pierre et Marie Curie - Paris VI, Groupe Hospitalier Pitié-Salpêtrière, 47 boulevard de l’Hopital, Paris 75013, France; 2 Center for Public Health Nutrition, University of Washington, Box 353410, Seattle, WA 98195, USA; 3 Nestle Waters France12 boulevard Garibaldi, Issy-les-Moulineaux 92130, France

**Keywords:** Water intake, Drinking water, Adequate hydration, Adults, Beverages, Dietary surveillance

## Abstract

**Background:**

Few studies have examined plain water consumption among US adults. This study evaluated the consumption of plain water (tap and bottled) and total water among US adults by age group (20-50y, 51-70y, and ≥71y), gender, income-to-poverty ratio, and race/ethnicity.

**Methods:**

Data from up to two non-consecutive 24-hour recalls from the 2005–2006, 2007–2008 and 2009–2010 National Health and Nutrition Examination Survey (NHANES) was used to evaluate usual intake of water and water as a beverage among 15,702 US adults. The contribution of different beverage types (e.g., water as a beverage [tap or bottled], milk [including flavored], 100% fruit juice, soda/soft drinks [regular and diet], fruit drinks, sports/energy drinks, coffee, tea, and alcoholic beverages) to total water and energy intakes was examined. Total water intakes from plain water, beverages, and food were compared to the Adequate Intake (AI) values from the US Dietary Reference Intakes (DRI). Total water volume per 1,000 kcal was also examined.

**Results:**

Water and other beverages contributed 75-84% of dietary water, with 17-25% provided by water in foods, depending on age. Plain water, from tap or bottled sources, contributed 30-37% of total dietary water. Overall, 56% of drinking water volume was from tap water while bottled water provided 44%. Older adults (≥71y) consumed much less bottled water than younger adults. Non-Hispanic whites consumed the most tap water, whereas Mexican-Americans consumed the most bottled water. Plain water consumption (bottled and tap) tended to be associated with higher incomes. On average, younger adults exceeded or came close to satisfying the DRIs for water. Older men and women failed to meet the Institute of Medicine (IOM) AI values, with a shortfall in daily water intakes of 1218 mL and 603 mL respectively. Eighty-three percent of women and 95% of men ≥71y failed to meet the IOM AI values for water. However, average water volume per 1,000 kcal was 1.2-1.4 L/1,000 kcal for most population sub-groups, higher than suggested levels of 1.0 L/1.000 kcal.

**Conclusions:**

Water intakes below IOM-recommended levels may be a cause for concern, especially for older adults.

## Background

Drinking plain water is an effective way to provide adequate hydration without calories [1,2]. Drinking plain water, tap or bottled, instead of caloric beverages, helps to reduce dietary energy density and may contribute to the management of body weight [3-8]. Water from beverages and foods is the key determinant of the energy density of the diet [9].

Adequate intakes (AI) for water are defined on the basis of three factors: observed water intakes in population groups, desirable water volumes per energy intake, and desirable osmolality values in urine or plasma [10-12]. The AI values for water from beverages and foods according to the US Institute of Medicine (IOM) are 2700 mL/day for adult women and 3700 mL/day for adult men [13]. These values were based on median intake estimates among younger adults from NHANES III.

The desirable water-to-energy ratio is another index of adequate hydration. In the US, the IOM Dietary Reference Intake (DRI) Subcommittee suggested the standard water requirement for adults at 1.0 L per 1,000 kcal of energy expenditure [13]. This value could be increased to 1.5 L/1,000 kcal, depending on activity level and water loss. Guidelines issued by the European Food Safety Authority (EFSA) [10] specify that the total available water intakes for adults should be no less than 1.0 L/1,000 kcal.

The established DRI values for water are based on water obtained from drinking water (tap and bottled); water from other caloric and non-caloric beverages, and on moisture from foods [10,13]. The DRIs were established by the IOM mostly to prevent the adverse effects of dehydration, and the IOM report indicates that considerable inter-individual variation exists in terms of necessary amounts of water to be consumed. Beyond issues of hydration, previous studies have shown that plain water consumption was associated with higher quality diets, better health behaviors, and lower risk for chronic disease in youth and adults [7,14-16].

With some exceptions [15,17,18], few studies have explored the consumption of plain water among nationally representative samples of US adults. To our knowledge, no studies have examined water intake using the most recently available dietary data. In addition, previous work has not broken down water consumption by beverage category. The present study was conducted using a large and nationally representative database: National Health and Nutrition Examination Survey (NHANES) 2005–2010 for adults ≥20y. Estimates of total dietary water from all sources (including plain water) from other beverages and from moisture in foods were compared to the IOM AI values. Additional analyses examined the contribution of different beverages to overall water and energy intakes. Lastly, we evaluated the water/calorie ratios (mL/1,000 kcal) and compared them to the recommended values.

## Methods

### Dietary intake databases

The present analyses used data from three cycles of the nationally representative National Health and Nutrition Examination Survey (NHANES), corresponding to years 2005–2006, 2007–2008 and 2009–2010. The National Center for Health Statistics (NCHS) has obtained IRB approval for all cycles of NHANES studies [19] and the data has been made available for public use via the NCHS website [20]. The three NHANES cycles provided us with a nationally representative sample of 15,702 adults age ≥ 20y.

These NHANES cycles were selected for two reasons. First, the collection of data on tap and bottled water consumed as a beverage only began in 2005 as part of the 24-h recall. In previous NHANES cycles, information about water as a beverage was not collected during the recall, but was assessed via questionnaire after the 24-h recall was complete. Second, the 2005–10 NHANES cycles included two 24-h recalls for most respondents, allowing for estimation of usual intakes using methods developed by the National Cancer Institute (NCI). The first recall was conducted by trained dietary interviewers in a mobile examination center while the second recall was conducted by telephone some days later [21-24].

### Plain water and beverage consumption

Beverages were classified into nine broad groups: Water (bottled or tap), milk (including flavored), fruit juice (100%), soda/soft drinks (regular and diet), fruit drinks, sports/energy drinks, coffee, tea, and alcoholic beverages.

The NHANES 24-h recalls for each respondent provide information on the amount in grams of each food and beverage consumed. All results presented are for mL of water content from selected beverages, not mean intakes by volume (e.g., we present mL of water in milk, not mL of milk consumed), as that information is not provided in the NHANES data.

### Energy intakes from beverages and foods

Energy intakes from different beverages and foods were estimated for each respondent. Food and beverage amounts were converted to calories (kilocalories [kcal]) using standard procedures for the United States Department of Agriculture Food and Nutrient Database for Dietary Studies.

### Statistical analyses

We used the National Cancer Institute (NCI) Method to characterize the usual intake distribution of total water, water consumed as a beverage (e.g., tap and water), and water and energy from beverage categories [25,26]. Two different approaches have been previously developed to estimate usual intake distributions using the NCI method: one for consumption of a ubiquitously consumed dietary component (e.g., calcium or total grains) and one for episodically consumed components (e.g., vitamin A or whole grains). The ubiquitous model fits a one-part nonlinear mixed model that incorporates only the amount consumed into the estimation of usual intake, while the episodic model fits a two-part mixed model that incorporates both the probability of consumption and the amount consumed in estimating the usual intake distribution. For total water, water from all beverages and water from food sources, the model appropriate for ubiquitously consumed dietary components was employed. All other values were estimated using the episodic model. For example, tap water was consumed by only 67% of respondents while less than half the respondents consumed bottled water on their first recall. For no beverage type and in no sub-population of interest did the frequency of consumption approach 90-95%, which would justify using the ubiquitous model. Additional covariates were included in the model to account for whether the recall data was from a weekday or weekend and whether it was the first or second recall [25,26].

In order to account for the complex survey design of NHANES data and estimate standard errors, balanced repeated replication (BRR) weights were constructed using WesVar software (Westat, Rockville, MD, 2012). A Fay’s adjustment of 0.7 was used and a total of 48 BRR runs were repeated for each analysis. The results are representative of the usual intake of the US population or sub-population of interest.

Because the NCI Method employs a random seed in running the models, values that would otherwise be expected to sum together may not do so (i.e., repeated runs of the same model can result in differences of ~1% between runs). Therefore, the sum of estimates of water or energy from specific beverage categories will not be expected to sum perfectly to the global estimate of total water intake. For example, the estimated mean value of total water consumed as a beverage was 1138 mL, while the estimate for tap and bottled water respectively was 644 mL and 502 mL, summing to 1146 mL. In estimating the population proportion of each beverage type to water and energy intakes, these estimates were obtained by dividing the category-specific value by the sum of all category-specific values. The population proportion is the percent total water or energy from specific beverage categories at the population-level. This measure can be interpreted as a ratio of the means, rather than a mean of the ratios, and is best suited for examinations of population-level dietary habits [27]. When the estimated relative standard error was greater than 30% for estimated means the results are not presented.

All analyses accounted for the complex survey design of NHANES and reflect the dietary behaviors of the US adult population from 2005–2010. The usual intake of water consumed as a beverage and total water were evaluated overall and by age group, gender, race/ethnicity, and family income-to-poverty ratio. The age groups were 20-50y, 51-70y, and ≥71y. Race/ethnicity was defined by self-report as non-Hispanic white, non-Hispanic black, Mexican-American, other Hispanic and mixed race/other. Family income-to-poverty ratios were defined as <1.0, 1.0-1.99, 2.0-3.49, and ≥3.

T-tests with unequal variances were used to test for differences in the mean intake level in each sub-group related to a reference group of interest. The reference groups used were age 20–50y, men, non-Hispanic whites, and those with a family income-to-poverty ratio ≥ 3.5.

All analysis used SAS software (Version 9.4 of SAS System for Windows, SAS Institute, Cary, NC, 2013) and estimates of the usual intake distribution used macros, code and methods adapted from NCI and the Centers for Disease Control (CDC) [28,29].

## Results

### Plain water consumption

Data presented in Table 1 show the consumption of plain water (total) in mL by age and by socio-demographic group. About 78% of adults reported consuming either tap or bottled water as a beverage on their first 24-hour recall.

**Table 1:** **Mean intakes**^**1 **^**of plain, tap and bottled water (mL) among adults by socio-demographic group**

	**n**	**Total water as a beverage**	**Pairwise p-value**	**Tap water**	**Pairwise p-value**	**Bottled water**	**Pairwise p-value**
All Adults	15702	1138 (16)	-	644 (13)	-	502 (13)	-
Age group							
20-50	8389	1294 (22)	ref	700 (17)	ref	597 (16)	ref
51-70	4737	1020 (20)	<0.001	607 (18)	<0.001	431 (14)	<0.001
≥71	2576	669 (12)	<0.001	495 (10)	<0.001	181 (9)	<0.001
Gender							
Men	7614	1153 (23)	0.30	660 (15)	0.12	509 (19)	0.73
Women	8088	1125 (14)	ref	628 (14)	ref	501 (12)	ref
Race/ethnicity							
Non-Hispanic White	7610	1134 (19)	ref	703 (17)	ref	437 (12)	ref
Non-Hispanic Black	3173	1129 (23)	0.87	513 (18)	<0.001	617 (27)	<0.001
Mexican-American	2899	1095 (25)	0.23	383 (22)	<0.001	729 (33)	<0.001
Other Hispanic	1322	1208 (41)	0.10	455 (35)	<0.001	758 (48)	<0.001
Other race – including mixed race	698	1314 (96)	<0.001	692 (60)	0.86	606 (37)	<0.001
Family income-to-poverty ratio							
<1	2905	1026 (33)	<0.001	603 (28)	<0.001	407 (21)	<0.001
1-1.99	3870	1088 (25)	<0.001	565 (22)	<0.001	542 (25)	0.27
2-3.49	3181	1115 (27)	<0.001	625 (22)	<0.001	505 (16)	0.86
≥3.5	4532	1223 (20)	ref	721 (17)	ref	509 (17)	ref

^1^Values are survey-weighted means with standard errors in parentheses.

On average, American adults consumed 1.1 L (1,138 mL) of water as a beverage per day. Older adults (≥71y) consumed less water than younger adults. Overall, men and women consumed comparable amounts of water as a beverage.

There was a strong effect of socioeconomic status on consumption of water as a beverage. Adults with higher incomes consumed more water as a beverage than adults with lower incomes. There was no marked difference by race/ethnicity, though the other race/mixed race group consumed the most water as a beverage.

Additional data from Table 1 show consumption of tap versus bottled water by age, gender, race/ethnicity, and family income-to-poverty ratio. Overall, adults consumed 644 mL/d of tap water (about 56% of total water consumed as a beverage) and 502 mL/d of bottled water (44%).

The patterns of water consumption varied strongly with age. For bottled water, consumption was strongly related to age; with younger adults consuming much more bottled water than older adults. The effect of gender on bottled/tap water consumption was not statistically significant.

There were strong socio-demographic effects on type of water consumed. Non-Hispanic whites consumed the most tap water and the least bottled water (703 mL/d from tap vs. 437 mL/d from bottled). By contrast, Mexican Americans consumed the most bottled water (729 mL/d from bottled vs. 383 mL/d from tap). Lower-income adults consumed 603 mL/d of tap water as opposed to 721 mL/d for higher income adults. There was a strong effect of family income on consumption of tap water. For bottled water, compared to adults with higher family incomes, only those with the lowest family incomes consumed significantly less bottled water.

### Water intakes from plain water, beverages, and foods

Table 2 summarizes the principal sources of total dietary water by age group. The principal beverage sources were plain water, soda, coffee, tea, milk, and alcohol, followed by fruit drinks and fruit juices. Since milk was often used with cereal, results are presented for milk (total) and for milk consumed as a beverage (i.e. not with cereal). Additional water was provided in the form of moisture from foods.

**Table 2:** Mean and percent of total water (mL) from various food/beverage categories by age group

	**20-50y**		**51-70y**		**≥71y**	
	**Mean (SE)**	**% of total water**	**Mean (SE)**	**% of total water**	**Mean (SE)**	**% of total water**
Water	1294 (22)	37.1	1020 (20)	32.0	669 (12)	30.1
Soda	466 (9)	13.4	312 (8)	9.8	140 (5)	6.3
Diet soda	164 (4)	4.7	178 (6)	5.6	62 (3)	2.8
Regular soda	310 (8)	8.9	135 (3)	4.2	79 (3)	3.6
Coffee	297 (6)	8.5	515 (11)	16.2	406 (8)	18.3
Alcohol	280 (9)	8.0	156 (6)	4.9	50 (3)	2.3
Milk	142 (4)	4.1	137 (4)	4.3	153 (4)	6.9
Milk (no cereal)	111 (4)	3.2	108 (4)	3.4	106 (3)	4.8
Tea	197 (5)	5.6	292 (7)	9.2	158 (6)	7.1
Fruit drinks	90 (3)	2.6	74 (4)	2.3	32 (2)	1.4
Diet fruit drinks	16 (2)	0.5	36 (3)	1.1	13 (2)	0.6
Regular fruit drinks	74 (3)	2.1	38 (3)	1.2	19 (2)	0.9
Fruit juice	74 (3)	2.1	59 (2)	1.8	59 (2)	2.7
Sports/energy	54 (3)	1.5	24 (2)	0.7	6 (1)	0.3
Water from food	590 (5)	16.7	598 (11)	18.3	547 (11)	24.4
Water from beverages^1^	2940 (20)	83.3	2665 (23)	81.7	1693 (15)	75.6
Total daily water	3563 (24)	-	3229 (27)	-	2251 (17)	-

^1^Milk consumed with food is included as a beverage.

The contribution of plain water, soda (regular and diet), alcohol and fruit drinks to water intakes tended to decrease with age. By contrast, the contribution of coffee and tea to total water intake increased with age. Older adults (≥71y) obtained about 18% of their daily water from coffee and coffee beverages compared to 8.5% among younger adults.

Among adults aged 20-50y, 83% of total water came from beverages, including 37% from plain water, and 17% from moisture in foods. For this age group, soda was an important source of dietary water, accounting for 13% of total water. Coffee and alcohol respectively provided 8.5% and 8% of total water.

Among adults aged 51-70y, 82% of total water came from beverages, including 32% from plain water, and 18% from moisture in foods. For this age group, soda provided 10% of total water, whereas coffee provided 16% and tea another 9%. Alcohol provided 5% of total water.

Among adults aged ≥71, 76.0% of total water came from beverages, including 30% from plain water. Twenty-seven percent of water came from moisture in foods. For this age group, soda provided 6% of total water, whereas coffee provided 18.0% and tea another 7%. Alcohol provided only 2.0% of total water for this age group.

**Figure 1:**
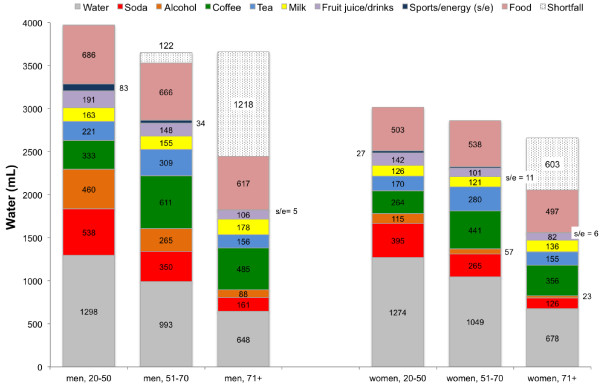
Water intakes from beverage/food category by age and gender among US adults.

Figure 1 shows the principal sources of total dietary water separately by gender and age group. For both men and women, the principal beverage sources were plain water, soda, coffee, tea, milk, and alcohol. The age-related decrease in soda and alcohol consumption and increase in coffee consumption were observed for both men and women.

The shortfall in water consumption relative to the IOM AI values for each age group is also indicated in Figure 1. It can be seen that as a group, older men failed to meet the AI value. The shortfall amount for that group was 1,218 mL/d for men ≥71. On average, women aged ≤70y exceeded the AI value, whereas women ≥71y had a shortfall of approximately 603 mL/d. Among adults 20-50y 42.7% of men and 40.6% of women failed to meet the IOM AI value for total water (3700 mL for men and 2700 mL for women). For adults 51-70y, 59.1% of men and 44.9% of women failed to meet the AI value for total water. Ninety-five percent (94.7%) of men and 82.6% of women aged ≥71y failed to meet the AI value for water.

### Energy intakes from beverages and foods

Table 3 shows the contribution to energy intakes from beverages and foods by age group. The beverages are separated by category. The contribution of foods to energy intakes rose with age, from 78.3% among younger adults to 86.3% among those ≥71y. The contribution of beverages to energy intakes declined from 21.7% among 20-50y to 13.7% among ≥71y. Soda accounted for 5.7% of energy intakes in the 20-50y age group but only 2.1% among those ≥71y.

**Table 3:** Mean and percent of total energy (kcal) from various food/beverage categories by age group

	**20-50y**		**51-70y**		**≥71y**	
	**Mean (SE)**	**% of total**	**Mean (SE)**	**% of total**	**Mean (SE)**	**% of total**
Milk	90 (2)	3.6	83 (2)	3.9	87 (2)	5.1
Milk (no cereal)	74 (2)	3.0	66 (2)	3.2	63 (2)	3.7
Soda	141 (4)	5.7	73 (2)	3.5	35 (2)	2.1
Alcohol	151 (4)	6.1	82 (3)	3.9	35 (3)	2.1
Fruit juice	39 (2)	1.6	31 (1)	1.5	31 (1)	1.8
Fruit drink	40 (2)	1.6	19 (1)	0.9	12 (1)	0.7
Tea	25 (1)	1.0	20 (1)	1.0	8 (1)	0.5
Coffee	8 (1)	0.3	8 (1)	0.4	6 (1)	0.4
Sports/energy	16 (2)	0.6	6 (1)	0.3	*	*
Water	-	-	-	-	-	-
Energy from beverages	526 (6)	21.7	351 (4)	17.0	225 (4)	13.7
Energy from food^1^	1901 (12)	78.3	1716 (12)	83.0	1419 (12)	86.3
Total energy	2437 (13)	-	2061 (14)	-	1643 (13)	-

*Relative standard error is greater than 30%.

^1^Milk consumed with food is included as a beverage.

### Water density per 1,000 calories

Total water intakes and water density per 1,000 kcal is shown in Table 4. The observed water volume per 1,000 kcal was between 1.2-1.4 L/1,000 kcal. Adults 50-70y, women, non-Hispanic whites and adults with higher incomes consumed the most water dense diets.

**Table 4:** Total water and water density among adults by socio-demographic group

	**Total H**_**2**_**0 from all sources (mL)**		**H**_**2**_**0 from all sources (mL) per 1,000 kcal**	
	**Mean (SE)**	**p-difference of means**	**Mean (SE)**	**p-difference of means**
All adults	3311 (19)	-	1369 (5.4)	-
Age group				
20-50y	3560 (30)	ref	1343 (6.9)	ref
51-70y	3229 (27)	<0.001	1442 (12.3)	<0.001
≥71y	2251 (17)	<0.001	1306 (11.7)	0.14
Gender				
Men	3779 (26)	ref	1274 (6.2)	ref
Women	2899 (16)	<0.001	1454 (9.3)	<0.001
Race/ethnicity				
Non-Hispanic White	3439 (24)	ref	1412 (8.9)	ref
Non-Hispanic Black	2854 (32)	<0.001	1185 (10.2)	<0.001
Mexican-American	3037 (36)	<0.001	1277 (9.8)	<0.001
Other Hispanic	3156 (44)	<0.001	1308 (11.1)	<0.001
Other race – including mixed race	3155 (67)	<0.001	1399 (16.8)	0.49
Family income-to-poverty ratio				
<1	3164 (37)	<0.001	1299 (11.4)	<0.001
1-1.99	3176 (27)	<0.001	1335 (11.3)	<0.001
2-3.49	3172 (30)	<0.001	1377 (12.6)	0.049
≥3.5	3512 (23)	ref	1411 (11.8)	ref

## Discussion

These analyses of total water intakes from all sources, including tap and bottled water, were conducted among a representative sample of US adults from the NHANES 2005–2010 database. The amounts of dietary water provided by plain water and by other beverages and foods were then compared to AI values by gender and by age group. The intent was to examine how close the population came to meeting the AI values, as defined by the IOM DRIs. According to the IOM, AI values may be used as goals for individual intakes though there is much inter-individual variation for water needs. Health status, physical activity or strenuous work, and environmental factors, such as temperature and humidity, are additional aspects to be considered when evaluating adequate intakes at the individual level [2,13,30-32].

A large proportion of older men (94.7%) and women (82.6%) failed to meet the IOM AI values. The average shortfall was 1218 mL (41.2 fl oz) for older men and 603 mL (20.4 fl oz) for older women. The average shortfall was only 122 mL (4.1 fl oz) for men 50–70, though 59.1% consumed less than 3700 mL per day. Although the average intake of water among women less than 70 was adequate; 45% and 41% of women 51-70y and 20-50y consumed less than 2700 mL of water per day. Younger men consumed the most total water, but 42.7% consumed less than 3700 mL of water per day.

The second criterion of adequate hydration, water volume (in mL) per 1000 kcal, did not fall short of desirable values, though such an evaluation is dependent on measuring energy intakes and expenditures accurately. Whereas the EFSA and IOM recommendations are at least 1.0 L per 1,000 kcal [10,13], the observed values of ~1.2-1.4 L/1,000 kcal were well above this cut-point, though there were some differences by population sub-group. Women, non-Hispanic whites and adults with higher family incomes tended to have diets that were more water dense.

The evaluation of water density suggests that water intake at the population-level is generally adequate, though evaluations of absolute values suggest that water intake may be too low among older adults. It is beyond the scope of this work to identify which of these two measures is the better indicator of water intake. Given the focus of the IOM DRIs Committee on the absolute intakes and the potential for under-estimating energy expenditure/intake, more emphasis should be placed on the absolute intake findings [13].

Biological markers, including serum or plasma osmolality, and to some extent urine osmolality, are additional markers of hydration status [10,33,34]. Until recently, hydration biomarkers were not included in NHANES data. In the 2009–2010 cycle, urine osmolality data were collected. Upon release of additional cycles that collect this data (to increase the sample size and availability of samples taken early in the morning), future work could examine patterns of this variable by population sub-group. However, no established cutoffs for hydration adequacy based on urine osmolality have been established at the population level.

The present analyses of the observed water intakes relative to the indices of hydration suggest that water consumption ought to be monitored more closely [35]. In 2010, EFSA published a 48-page report on water consumption, arguing that water is often disregarded in national and international recommendations or is very cursorily treated [10]. For example, the 2010 US Dietary Guidelines Advisory Group report devoted only two pages to water, stating that most healthy people consumed adequate water to meet their needs. Because water needs vary considerably, they concluded that a minimum intake of water could not be set [36].

The current study was unique in focusing on the consumption of plain drinking water and other beverages using the most recently available data for American adults. Previous work evaluating beverage intakes of adults has focused on the predictors and correlates of consuming specific beverages [37-40], the relation between beverage intake and measures of diet quality [13,15,40], the contribution of beverages to nutrient or energy intake [41,42], or time-trends in beverage consumption patterns or preferences [43,44]. One important finding from the current study was differences in tap vs. bottled water consumption by socio-demographic factors, namely race/ethnicity, but also by family income. A recent study focused on the relation between the perceived safety of tap water and the intake of sugar sweetened beverages among US adults, nothing that those who viewed tap water as safe to drink tended to be older, have higher incomes, be better educated, were more active and were more likely to be white [45]. Water safety has previously been raised as a concern and may explain the higher proportion of Mexican-American and other Hispanics who consume bottled vs. tap water [46,47]. However, none of these studies measured water consumption directly. It is unclear why a weaker preference was observed for the non-Hispanic black population in the present study.

Another recent report, based on the 2007 National Cancer Institute’s Food Attitudes and Behaviors Survey, examined behaviors and attitudes associated with low consumption of plain water among US adults [48]. Here, the adjusted odds of drinking <4 cups of water per day was associated with older age (>55y), sedentary lifestyles and low consumption of fruits and vegetables, but not with education or incomes. However, in this study regular water consumption was reported via questionnaire, not by measurement in a dietary recall or interview.

The present study therefore fills a gap in the existing knowledge regarding water consumption patterns among US adults. Although older adults are known to represent a group at risk, current data on water consumption patterns have not been available previously.

Future guidelines on beverage consumption should take plain drinking water into account. This is particularly important given the size of the shortfall between observed intakes and IOM AI values for older adults. This is particularly important given the increased likelihood of having an impaired thirst mechanism among older adults [49].

Total water intake can be increased in a number of ways. The most effective way would be to increase the consumption of plain water, including either tap or bottled water. Promoting water intake is currently highlighted in the 2010 Dietary Guidelines for Americans as a potential replacement for sugar-sweetened beverages [50]. Future dietary surveillance should monitor total water intake to determine if reducing intake of sugar-sweetened beverages has a negative impact on total water intake.

In the present analyses of NHANES 2005–2010 data, non-beverage food sources accounted for 17-25% of total dietary water, as compared to 19% reported in the 2010 Dietary Guidelines for Americans Advisory Committee report [36], though methods for assessing water intake have changed from previous NHANES cycles. This observation highlights that increasing consumption of low energy density foods with high water content foods (e.g., fruits/vegetables) is another approach to increase water intakes, while subsequently improving overall diet quality.

The present analyses had some limitations. First, the NHANES data are based on self-report and are subject to random and systematic reporting errors. Each of the two dietary recall days used different methods to collect the data, which may introduce mode effects into the estimate of water consumption. If water intakes were under-reported in the NHANES database, then the estimates presented here will over-estimate the percent of adults who fail to meet the recommended intakes. It is probable that many respondents under-reported water intakes due to drinking water lacking salience. This may be particularly problematic for events where little water was consumed or it was consumed casually (e.g., repeatedly being refilled at a restaurant). It is important to note that these data cannot be directly compared to those from pervious cycles of NHANES (prior to 2005), as the mode for collecting data on water intake changed. In previous cycles of NHANES water intake was measured at the end of the recall via questionnaire, whereas in more recent cycles, water is measured as part of the 24-hour recall. Comparisons of water intake for the entire population and population sub-groups between 1999–2004 and 2005–2006 reveal that estimated water intakes are approximately 15% lower using newer as compared to older data [15]. While this difference may be attributable to secular changes in water intake, they are more likely driven by changes in data collection. Caution should be applied when comparing the results presented here to data collected prior to 2005. An additional limitation in evaluating adequacy of water intake at the population-level is the lack of Recommended Daily Allowance values for water. While the AI values established by the IOM provide some benchmark in evaluating water intake, the proportions above/below this value should be interpreted cautiously. The 2010 Dietary Guidelines for Americans contends that the combination of thirst and normal eating/drinking behaviors provides sufficient water [49]. Surveillance of water intake from dietary data should be carefully monitored and the use of biomarkers to evaluate hydration status at the population-level should be a priority.

Nonetheless, the present analyses represent one of the few explorations of the consumption of water in the US and can be used to inform approaches to improving the overall diet quality and hydration status of the population. Advantages of the data used here include the use of a large and nationally representative dataset that forms the basis for dietary surveillance in the US.

## Conclusions

Among older men and women, there is evidence of inadequate water consumption in absolute terms. Fewer than 4.3% of men and 17.4% of women aged ≥71y consumed the recommended amounts of total water. Increasing total water consumption can be achieved through various means, though promotion and encouragement of non-caloric beverages is likely to be the most successful avenue for increasing water consumption without increasing energy intakes.

## Abbreviations

DRI: Dietary reference intake; NCI: National Cancer Institute; NHANES: National Health and Nutrition Examination Survey; IOM: Institute of Medicine; EFSA: European Food Standards Agency; AI: Adequate intake.

## Competing interests

The authors declare that they have no competing interests.

## Authors’ contributions

AD and CDR designed the study. CDR analyzed the data. All authors and participated in drafting the manuscript. All authors read and approved the final manuscript.

## Pre-publication history

The pre-publication history for this paper can be accessed here:

http://www.biomedcentral.com/1471-2458/13/1068/prepub
